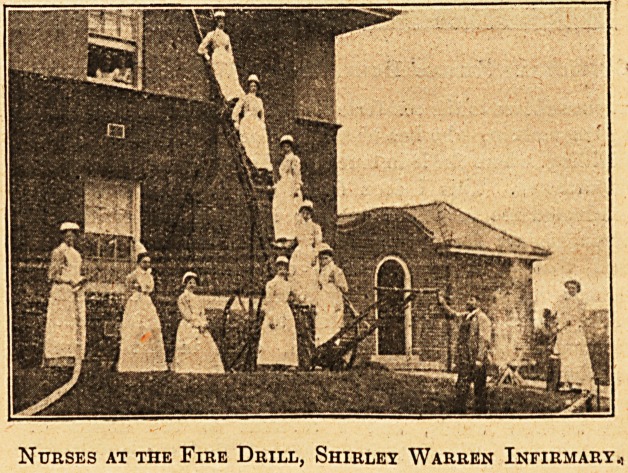# The Hospital. Nursing Section

**Published:** 1907-03-23

**Authors:** 


### The Hospital.

**Nursing Section.**

Contributions for "THE HOSPITAL," should be addressed to the EDITOR, "THE HOSPITAL" NURSING SECTION, 28 & 29 Southampton Street, Strand, London, W.C.

No. 1,071.—VOL. XLI SATURDAY, MARCH 23, 1907.

### Notes on News from the Nursing World.

### A NEW ZEALAND DOCTOR ON ENGLISH HOSPITAL MATRONS.

DR. EWART, medical superintendent of Wellington Hospital, New Zealand, has been giving, in some of the New Zealand papers, an account of a visit which he recently paid to England. He has a great deal to say about English hospitals, which he compares unfavourably with those in his own colony. Judging by the reference he makes to the relations existing between doctors and matrons in this country, his knowledge of English hospitals must, however, be limited to institutions of a quite unusual character. He affirms that in England the matron is "altogether independent of the doctor," and in "many cases she would countermand his order." We do not object to Dr. Ewart's conclusion that, because he saw nurses from New Zealand in London, and "heard" of two more in the provinces, New Zealand nurses are in special demand at home; but his picture of responsible English hospital matrons "countermanding" the instructions of the medical men is a proof that his sources of information are strangely unreliable.

### "THAT QUESTION OF UNIFORM."

"TRUTH," of March 13, mentions a letter from a nurse who complains of the discredit her profession suffers from the doings of those whom she describes as "wolves in sheep's clothing—adventuresses posing as nurses." *Truth* points out that nurses are not the only people who are thus aggrieved, and mentions the theatrical profession and journalists, who often blush for shame at the frequency with which their callings are similarly "besmirched by all sorts of shady characters." As to the question of uniform, *Truth* holds that the wearing of uniform by nurses may be "attributed to a little feminine vanity, for the dress is really attractive," a fact of which "the legitimate nurses are perhaps not altogether unconscious." *Truth* suggests one method by which nurses can guard against a wrong using of their professional costume. This is the adoption of a uniform that is "ugly" or "at least dowdy," but *Truth* fears—and we are inclined to agree with the writer—"that most nurses will regard this remedy as worse than the evil it is designed to cure."

### THE ADVANTAGES OF A MONEY DIET.

IN the account given on another page by our Commissioner of his visit to Shirley Warren Infirmary, one of the most up-to-date of poor-law training schools, the matron informed him that for the old system of rations has been substituted a money diet. She states that among the advantages of the new system is that there is no waste; and obviously it cannot be more expensive because, if the amount allowed in money is exceeded, the steward, acting as representative of the Southampton Guardians, cuts the menu down. There is also the considerable advantage that instead of the nurses having little variety of food, they get an excellent choice. The diet table for the week, which the matron handed to our representative, is all that any reasonable person in any institution could expect; and if the catering and the manner in which the meals are served be equally satisfactory, the nurses at Shirley Warren have no cause for complaint in respect to the catering. Administrative difficulties are in the way of the adoption of this plan in many Poor-law infirmaries where it might prove equally popular.

### "EXCEPT IN NURSING."

IT is noteworthy that the candidates for the office of Poor-law Guardians, who are supported by the London Reform Union for election, pledge themselves to use pauper labour as much as possible, "except in nursing." The exception is one of great importance, and we desire to emphasise it by expressing the wish that candidates everywhere will promise to do their utmost absolutely to abolish pauper labour in the nursing of the sick. It is not only bad in itself, but it fails to promote economy.

### INFIRMARY NURSES LIVING IN LODGINGS.

THERE was great rejoicing last week among the nurses of the Plymouth Poor-law Infirmary because the foundation-stone of the new building at last was laid. Under existing conditions most of the members of the nursing staff live out in lodgings, and it can be understood that they will welcome the early completion of suitable quarters in the new building. It is 15 years since the Guardians first took up the matter, so that they have not even made haste slowly. The superintendent nurse must have found it increasingly difficult to fill vacancies. When the bad arrangement of sleeping out comes to an end Plymouth Infirmary will become more attractive to intending probationers than it has been in the past.

### A NOVEL NURSING SCHEME.

A MOVEMENT has been started in Scotland called the Trained Nurses' Visiting Guild. Each branch is to consist of ladies, is to be limited to 500 members, and each member is to pay an annual subscription of five shillings. This will entitle the member to the services of a nurse whenever she is required. The total sum, it will be seen, is £125 per year, which, we agree, is enough to pay the salary of a single nurse and leave something over for working expenses. The movement is evidently intended to meet the needs of the middle-classes whose means are limited, and we hope that in practice it will work out happily.

### THE GUILD OF SERVICE.

We publish elsewhere a report of a meeting which was held at the Church House last Friday in order to form a Guild of Service. The movement will interest many of our readers, because it is sought to enlist the support of all nurses and officers connected with poor-law institutions. One of the reasons given for joining the Guild is that nurses resident in great institutions and in mental asylums "are liable to fall outside the range of the ordinary parochial agencies of the Church"; and it is said that "instances are known of good Churchpeople coming to a Union in a neighbourhood where they had no friends and lapsing from their religious duties in the saddest way." If by the instrumentality of the Guild of Service the want can be supplied and the danger avoided, the new organisation will justify its existence. We are disposed to agree with those who think that there is more danger in these days of the spiritual than of the material starvation of nurses; but the assertion of one of the speakers at the inaugural meeting of the Guild that poor-law nurses and workers have hitherto been "almost forgotten by the Church" is, surely, an exaggeration.

### "THE THANKSGIVING BICYCLE."

A novel mode of obtaining a bicycle for a district nurse is described by the author of the "Incident" in our current number. There are cases in which it may be difficult to find the needful money to enable the nurse to visit patients in outlying districts; and in such the difficulty might be solved in the same manner as in this instance. We publish the article now in order that if the idea is welcomed it may be adopted in the autumn.

### ANOTHER NURSES' REGISTRATION BILL.

Mr. Munro-Ferguson has again introduced into the House of Commons a Bill "to regulate the qualifications of trained nurses and to provide for their registration." It consists of twenty-six clauses, and is backed by Sir John Dickson-Poynder, Mr. Rose, Mr. Rainy, Mr. Eve, Mr. Fell, Mr. Crooks, Sir George Robertson, Dr. Rutherford, Mr. Jowett, and Mr. McKillop.

### THE STEAM KETTLE IN INEXPERIENCED HANDS

A catastrophe which has just occurred at Cardiff in consequence of the use of a steam kettle by an inexperienced person merits attention. The victim, a child of seven months, suffered from bronchitis and croup, and the medical man advised that it should be treated by being enclosed in a tent-like compartment, placed alongside the fire, and that steam from a boiling kettle should be conducted into the compartment by means of a funnel. The mother made a paper funnel, and steam was proceeding through it when the mother left the room for a couple of minutes. Upon her return she found the paper funnel bent and the steam playing on the child's face. The child was scalded so badly that she died the same day. The medical man, in explanation at the inquest, said he directed that the child should be steamed, but not towards the head. A scald, or a burn, is, however, so quickly developed, so far-reaching, and so often fatal in its effects, that the use of the steam kettle should only be recommended when the services of a district nurse are available to arrange the tent.

### TWO NOTABLE APPOINTMENTS.

The Board of Management of the London Homœopathic Hospital on Thursday last selected Miss Clara Hoadley as matron in succession to Miss Victoria Daunt. Miss Hoadley, who was one of a large number of applicants, was trained at Guy's Hospital, and holds the silver medal. Since her training she has been assistant matron at the Royal Infirmary, Preston, for two years, lady superintendent and matron of the Royal National Hospital for Consumption in Ireland, and from 1904 matron of the Coventry and Warwickshire Hospital. The vacant post of matron of Wandsworth Union Infirmary was filled, on the same day, by the appointment of Miss Helen Todd, matron of the Royal National Sanatorium, Bournemouth. Miss Todd was trained at St. Bartholomew's Hospital, and her Poor-law experience was gained at the Hackney Union School at Brentwood, of which she was assistant matron for nine months. She has also temporarily acted as matron, and on several occasions as midwifery superintendent at the Brighton Hospital for Women. She leaves a very pleasant, to undertake a very arduous, post.

### THE NEW MATRON OF EDINBURGH ROYAL INFIRMARY.

For the coveted post of Lady Superintendent of Nurses of Edinburgh Royal Infirmary there were 18 applications—one from America, fourteen from England, and three from Scotland. At the weekly meeting of the Board on Monday Miss Annie Warren Gill, R.R.C., was appointed successor to the office which Miss Spencer will vacate on June 30. The other selected candidates were the matron of the Royal Edinburgh Hospital for Sick Children, the matron of Dundee Royal Infirmary, and the matron of the Birmingham General Hospital. Miss Gill, who has been matron of the Royal Berkshire Hospital since June 1903, was not only trained at Edinburgh Royal Infirmary, but worked her way up to the position of assistant matron, and she therefore now becomes, after varied service, including valuable work in South Africa, which was recognised by the Imperial Government, head of the school with which she has been associated during the greater part of her career.

### CHILDREN AS COLLECTORS.

The annual meeting of the South London District Nursing Association was held at Clapham Rectory on Saturday last week. A feature of the report, to which reference was made, was that by means of collecting cards £61 had been collected. Several of these cards were taken by children who, in some cases, did remarkably well in obtaining a number of small sums. Four of these were invited to the meeting, and afterwards partook of tea, which they evidently greatly enjoyed. Among other contributions was £70, the proceeds of tableaux organised by the superintendent, Miss Bullock, and £15 from the Wandsworth and Clapham Guardians. The speakers included the Rev. A. B. Peile, Master of St. Katharine's Hospital, Canon Erskine Clarke, Mr. W. H. Dickinson, M.P., and Mr. D'Arcy Power, F.R.C.S. Each bore testimony to the work of the nurses, who paid over 40,000 visits in the year, and, amongst other duties, examined nearly a thousand children on behalf of the Country Holiday Fund. Canon Clarke made a point when he alluded to the nurses whom, he said, he often met flying round on their daily visits, as "incarnations of energy and vigour."

### GUY'S HOSPITAL MUSICAL SOCIETY.

On Tuesday evening Guy's Hospital Musical Society had their second and last concert of the season in the Court Room. The music was entirely sacred, and consisted chiefly of selections from works of Mendelssohn. Several nurses and students contributed excellent solos. A hearty vote of thanks was passed to Mr. H. Taylor, the conductor, and others who had helped to make the evening a success.

### THE GOVERNMENT AND THE MIDWIVES ACT.

On Friday at noon this week a representative deputation, arranged by the Association for Promoting the Training and Supply of Midwives, will be received by Lord Crewe at the Privy Council Office. The object of the deputation is to lay before the Lord President of the Council certain points in connection with the proposed exemption of Poor-law midwives from the rules of the Central Midwives Board, and the reply of the Lord President can scarcely fail to have an important bearing on the situation.

### THE MAJORITY OF A NURSING ASSOCIATION.

The annual meeting of the Southport and Birkdale District Nursing Society, which has just been held, was exceptionally interesting as the organisation has just attained its majority. When it was started, twenty-one years ago, it was a very modest undertaking, and for some time its income was only between £200 and £300 a year. Last year the total receipts amounted to £981 13s. 1d., of which £553 12s. 11d. was derived from subscriptions and donations. The needs of the place have, however, increased correspondingly, and the balance in hand at the end of the year was less than £10. We agree with Sir George Pilkington, who was one of the speakers at the annual meeting, that the people of Southport, with all their wealth, might do better than this, and that, since more nurses are wanted, the income ought considerably to exceed £1,000 a year. Meanwhile, we are pleased to note that the new branch of work, towards which the Society receives a grant of £50 from the borough funds, is described by the medical officer of Southport as very satisfactory; and that the nurse, whose duty it is to look after and watch over the infants born in the district during the first year of their existence, has proved an important addition to the staff.

### THE IRISH NURSES' ASSOCIATION.

The fourth anniversary of the Irish Nurses' Association was held at 86 Lower Leeson Street, Dublin, on Saturday evening last. The chair was taken by Mrs. Kildare Tracy in the absence of the President. After the minutes of the last annual meeting were read, Miss Hampson, Miss Huxley, and Miss Shuter were elected to act as the Finance Committee. Mrs. Tracy then read an address from the outgoing President, Miss Kelly, lady superintendent of Dr. Steevens' Hospital, which contained a *résumé* of the year's work, and included many events of importance. Miss Ramsden was elected Vice-President for the ensuing year by a large majority. Mrs. Kildare Tracy, lady superintendent, City of Dublin Institute, then took the chair as President, and a hearty vote of thanks to the retiring President was proposed in a few well-chosen words by Miss Huxley, and seconded by Miss Lamont, superintendent of the Irish Branch of Queen Victoria's Jubilee Institute, and carried by acclamation. A special welcome was given to Lady Hermione Blackwood, President of the Ulster Branch of the Association, who expressed much pleasure at being able to attend. Mrs. Kildare Tracy made mention of the regret of the Association at losing the valuable services of their Secretary, Miss M. E. MacDonnell, owing to her departure to the mission-field in India, and drew attention to the badge of the Association, which might be had at any time from Miss Cherry, the newly-elected Secretary. At the conclusion of the business meeting tea was served, and the usual pleasant social evening enjoyed.

### NURSING SISTERS IN ROME.

We so frequently receive inquiries respecting the provisions for the nursing of the sick in Rome that the extension of the work of the community of nursing sisters known as "The Little Company of Mary" will excite interest. Lady Herbert of Lea states that the sisters, who have been at work in Rome for a quarter of a century, find the demand for their services so great that they have acquired a considerable property on the Celian Hill, near the outskirts of the city. Here they have raised a more commodious building, which is about to be opened. Patients are to be received and attended without distinction of creed, an[illegible] there is no fixed scale of remuneration for the nursing, the visitors attended being at liberty to make an offering according to their ability.

### SHORT ITEMS.

Lord Portman has contributed £1,000 to Queen Charlotte's Hospital for the enlargement of the Nurses' Home.—As a train was arriving at West Drayton on Sunday morning from Paddington a gentleman in the charge of a nurse suddenly jumped in front of the engine and was cut to pieces. He had been an inmate of a private asylum in the locality, and had gone to the station in order to meet a relative.—A special series of meetings for nurses, under the auspices of the Nurses' Missionary League, will be closed on Saturday with an address, at 7 p.m., by Mr. R. Wilder, M.A., to be delivered, by kind permission, at 1a Adelphi Terrace, Strand.

### The Nursing Outlook.

"From magnanimity, all fears above;
From nobler recompense, above applause,
Which owes to man's short outlook all its charm."

### THE COST OF A DISTRICT NURSE.

FEW people, if we may judge from the advertisements which appear from time to time, seem to have any true idea of the expenses which a district nurse has to meet, of the cost of her maintenance, of the importance of affording her every facility for the continuous maintenance of good health, or of the extras, apart from salary and house room, with which she should be provided at the cost of the community to whose needs she may minister. In starting a district nurse and making arrangements for the nurse's rooms it is well to do so provisionally, so as to enable a change to be readily made if found desirable. The district nurse should reside near the centre of her work and her rooms should be located in a convenient position. Where a house is provided it is essential that a servant should be supplied. Queen Victoria's Jubilee Institute, after many years' experience, lays down the rule that the nurse should not be allowed to live alone. If a mother or other relation can live with her, many difficulties are thus solved. Where such an arrangement cannot be made the nurse should be placed with some one responsible for cooking her meals, for otherwise, too often, no proper food is prepared and the nurse's health suffers. This is natural enough when it is realised that in country districts the nurse's work may often be very irregular, especially if she be engaged in midwifery. Such a nurse may be needed almost day and night for a week or ten days, and then have very little to do for some time. These general considerations are too frequently not realised or recognised, and so avoidable trouble arises.

Dealing next with the nurse's emoluments, it should be clearly understood that a Queen's nurse, and indeed any really capable and fully trained district nurse, cannot be fairly remunerated and properly maintained for a less sum than from £85 to £100 a year. The minimum salary is from £30 to £35 per annum, with an annual allowance of £4 for uniform and in addition allowances for laundry, fire and light; quite apart from local conditions, each Queen's or district nurse should further be provided with board, furnished rooms, and attendance. These expenses can be met by paying the nurse £90 a year and allowing her to provide everything for herself, or by paying her the salary just named and providing her with all the other items we have enumerated. Thus, the cost of board cannot be estimated at less than 10s. a week, laundry 2s. a week, the cost of two furnished rooms, with light and attendance, being governed by local rates. In some localities lodging, fuel and food are one or all specially expensive, and, in such a case, £85 for the commencing salary is not enough. The nurse has further to be supplied with certain books and appliances, and a suitable cupboard in which to keep them, and other things which are necessary in her work. Sometimes the district supplies the nurse with a bag or bags, but usually the nurse possesses her own equipment in these respects. A further outlay is entailed by the provision of a bicycle or some other conveyance, the cost of which must be supplied by the local committee.

One of the greatest difficulties which has beset the full development of district nursing has arisen from the fact that, many of the associations can hardly gather the required amount for the nurse's maintenance, and therefore are unable to increase the salary for nurses of greater experience. Every community will do well to bear in mind that, it is not possible, for any woman, without private means, to live properly, even in inexpensive country districts, for less than £85 per annum.

Eloquent testimony is borne to the value attached to the work of a district nurse by the widespread employment of these women in recent years throughout the country. In scattered districts it is often impossible to obtain sufficient funds to maintain a Queen's nurse. In such cases the Institute has undertaken to affiliate country nursing associations under special conditions. These conditions require that all village nurses, employed by an affiliated county association, shall have, if possible, twelve months' training, and must be registered midwives under the Midwives Act. Village nurses are usually women of the community, and the expenses of their training in midwifery and district nursing have been frequently defrayed by special grants from the County Councils. In return for this training the nurses are bound by agreement to serve the county association for a period of three years at a lower salary. A village nurse costs the local association from £45 to £60 a year, including salary, board, lodging, and uniform. To ensure efficiency the Institute provides that all such village nurses shall be under the direct supervision of a local superintendent, who is herself an experienced Queen's nurse, and a portion of whose salary is paid by the Institute. When the residents in any county desire to form a county association, they have first to co-operate to raise a central fund to train county women as village nurses, to maintain a county superintendent, and to extend district work throughout the county. This work is very much simplified by the excellent organisation now accomplished by the Institute, which supplies on application suggested constitutions, by-laws, and other necessary information. Over 700 associations employing Queen's nurses are now affiliated to the Institute.

### Nursing in Tropical Climates.

By ANDREW DUNCAN, M.D., B.S., M.R.C.P., F.R.C.S., Fellow King's College, Lecturer on Tropical Medicine at the London School of Tropical Medicine, and the Westminster Hospital.

### VIII. DYSENTERY.

(*Concluded from page* 337.)

### THE NURSING OF A CASE OF DYSENTERY.

Should the signs of dysentery become apparent in a patient, no time should be lost in summoning the doctor. Before the latter arrives it will do no harm to wash out the bowel with an enema of warm water, to a couple of pints of which two teaspoonfuls of boracic acid may be added. The main principles of treatment will be (1) rest, (2) diet, (3) drugs. The latter need not detain us, as they are the especial province of the physician.

### 1. REST.

The dysenteric patient must at once be put to bed. Rest must be complete, as by this the movements of the intestine are controlled. The use of the bed-pan is essential. Fomentations should be applied to the abdomen. Professor Maclean, of Netley, used to advocate the use of a water-belt, whilst Cantlie recommends a large thick pad, broad enough to cover the whole front of the abdomen. This pad may be either wet or dry.

### 2. DIET.

(*a*) *In the acute form* this should be as small in bulk as possible and liquid in character, and consist of milk, with whey or ordinary weak tea. Whey is made by taking half a pint of fresh milk heated lukewarm (115° F.); add one teaspoonful of Fairchild's essence of pepsin, and stir just enough to mix. When this is firmly coagulated, beat up the curd with a fork until it is finely divided, and then strain. Lemon-juice or sherry may be added to flavour it. If the thirst is severe the patient can suck small pieces of ice. Where milk cannot be taken by the patient strong beef-essence is indicated. If he be very weak give him some form of alcohol. As the condition improves rice cooked in milk or broth or gruel of tapioca may be given, and so gradually the diet increased. Solid food must not be taken till some days have elapsed after the disease has abated. All the food must be given in small quantities at a time, and none of it should be taken hot. I remember the very sad case of a brother officer not long after my arrival in India. He had successfully passed through an acute attack of the disease and was nearly convalescent. He was staying in the house of a mutual friend, who left him alone for a few hours. The patient, whose appetite was now somewhat urgent, took advantage of his friend's absence, and left the house to purchase some food. Unfortunately he chose a tin of herrings, which he devoured. Two days afterwards he had succumbed to an attack of gangrenous dysentery.

For natives Buchanan recommends milk (fresh or curdled), rice-water, sago or arrowroot, or "mar" or "dahi" (eight ounces three times a day). "Mar" is made by boiling one pound of fine cleaned rice with three pints of water, and straining—a white starchy substance of the consistence of porridge—will result. "Dahi" is "tyre" made from milk. The two are mixed together and eaten at 10 A.M., 5 P.M., and 7 P.M.

(*b*) *In chronic dysentery* the diet must be more liberal, as the patients are weak and anæmic; but at the same time it must be simple and easy of digestion. Tender chicken, pounded meat, strong soups are indicated, with toast well soaked in cocoa. Egg-flip is also excellent.

### PREVENTION OF THE DISEASE.

(*a*) As regards the immediate prevention of the disease from infection by the patient, the nurse should thoroughly disinfect the stools before they are disposed of, either by means of one of the disinfectants mentioned in a previous lecture, or by boiling, or, lastly, by burning them. The addition of petroleum, as recommended by the late Professor De Chaumont, is an excellent plan if this last method be carried out. In the case of dysentery arising amongst a large body of individuals as well as in isolated cases, the excreta must be protected from flies. This can be effected by pouring chloride of lime on them, as it form a protecting layer, and its odour keeps off the flies. Keating's powder dusted over the walls of the room into which the motions are removed for treatment before burial has also been found efficacious in keeping off flies. The bedding must be also disinfected after use.

(*b*) *The precautions to be taken by the nurse herself* consist in her being careful as regards her diet. All indigestible articles of food must be avoided and her water should be filtered. As regards her dress, she should always wear her cholera belt, and she should never allow herself, if possible, to become chilled. Cotton should not be worn next the skin, but light merino. Various prophylactic drugs have been recommended, but the evidence in their favour is not striking; thus, in the French Tonquin Expedition, bismuth was given to the troops. In the China War of 1860 and in the Malay Expedition of 1875-76 quinine was administered. Lastly, sulphuric acid has been recommended with a view of destroying the micro-organisms. If any are taken, bismuth is certainly not advisable, as this would tend to constipation, and constipation predisposes to dysentery.

### To Nurses.

WE invite contributions from any of our readers, and shall be glad to pay for "Notes on News from the Nursing World," "Incidents in a Nurse's Life," or for articles describing nursing experiences at home or abroad dealing with any nursing question from an original point of view, according to length. The minimum payment is 5s. Contributions on topical subjects are specially welcome. Notices of appointments, letters, entertainments, presentations, and deaths are not paid for, but we are always glad to receive them. All rejected manuscripts are returned in due course, and all payments for manuscripts used are made as early as possible after the beginning of each quarter.

### The Care of the Throat, Nose, and Ears in Children.

By St. Clair Thomson, M.D., F.R.C.P. Lond., F.R.C.S.Eng., Physician for Diseases of the Throat in King's College Hospital.

*(Continued from page 352.)*

The appearance of the habitual mouth-breather is now so well known that we are able to recognise him across the street. The victims of nasal obstruction are seen to have a narrow, undeveloped nose, a drooping and projecting lower jaw, a constantly open mouth, through which the upper teeth project, while the absence of action of the muscles round the mouth give to the face a smooth, stupid, vacant, semi-idiotic expression. These patients are apt to be round-shouldered, with a tendency to curvature of the spine, narrow-chested, and not infrequently pigeon-breasted. Such is the appearance of the adenoid child with whom we are all acquainted nowadays. Adenoids are no new invention, nor are they limited to any race or climate. Photographs show that they are known from Greenland to the equator, while pictures in the Florence Galleries and statues in the Vatican prove that they existed in the periods of the Renaissance and of Greek antiquity.

Now that we have studied the natural methods of defence in the upper air-passages, we are in a position to think over the care to be taken of them in childhood. First of all, both for the sake of general health, and not to overwork a willing servant, the air supplied to the body should be as pure as possible. Although climates have their advantages, we must remember it is more important how we live than where we live. Sewage air should be as much avoided as contaminated food and drink. We have seen how the nose filters and warms the air before it reaches the lungs, so that no one at any age need fear an abundance of outside air. At night, so long as the child is warm in bed, the night air may blow over it freely. When it takes its walks or drives abroad it need not be half-smothered in a thick veil, with the hood of the perambulator up and backed against the wind, so that it breathes over and over again its own exhalations. Respirators over the mouth are useless, if the mouth is closed, for the nose is Nature's perfect respirator. If mouth-breathing is a necessity the respirator starves the wearer of his much-needed oxygen.

Superabundant mucus from the nose must be got rid of. For man the natural and civilised method is by blowing. No child instinctively knows how to blow its nose, just as no child knows how to expectorate. The expectoration which a child raises from its chest it promptly swallows into the stomach, and any excess of mucus from the nose is allowed either to stagnate there, to flow backwards into the throat, or to dribble on to the upper lip. At the best it is simply wiped away—sometimes by the nurse with her own already contaminated handkerchief. The nose should be blown out, and not simply have its extremity dabbed. In the art of cleansing the nose the man in the street is the sensible person. One nostril should be closed with the thumb (covered with the pocket handkerchief), while down the other nostril a full blast of air from both lungs drives all secretion into the handkerchief. The process is then repeated for the opposite nostril. Children with cararrh should have their nostrils thoroughly cleared in this manner two or three times a day, and not be ineffectually irritated by simply having their noses frequently wiped.

Vigorous exercises—running, dancing, skipping, and all sorts of games—should be carried out with the mouth closed. But if the child has difficulty in breathing through the nose it is not wise to insist on the closed mouth. Ineffectual efforts to draw sufficient air from a narrow nose may result in the indrawing of the chest walls, as seen in the photographs I hand round. It is in such cases that the so-called breathing exercises do harm; their use comes in after the nose has been rendered normally patent.

For the care of the lower throat—the pharynx as we call it—there can be no doubt that the act of mastication has a healthy influence, and children should be taught to chew their food properly. Mufflers round the neck should be abolished; they do not reinforce any of the natural defences, but rather weaken them by interfering with the physiological action of the skin.

The proper use of the voice is the best protection for the vocal cords. The subject of voice use, including breathing, vocalisation, and enunciation would lead us into too wide a subject at present. We English, as a race, are such bad speakers that we should lay to heart Carlyle's remark where he says that "God has gifted you with a tongue and has set it between your teeth that you may show to us your true meaning, not that it should be rattled like a muffin-bell." In children it is remarkable what an amount of, to us, high-pitched, screaming, and yelling their little throats will stand. We should not check it, for joyous shouting and laughing, particularly in the open air, is doubtless a more natural and healthy exercise than any amount of informal indoor breathing-exercises. But we should take particular note if the child's voice appears to have at all what we call a nasal tone. This term is given when the nose is more or less obstructed. Of course as a matter of fact the voice does not assume a nasal character—it is just the want of nasal resonance which gives to the voice this dead, smothered character. The method which I employ is the use of the words, "Clapham Common." If a child pronounces it "Glabbad Gobbod," we are generally right in suspecting some nasal obstruction, and most probably adenoids.

Hearing is probably of all the special senses the most important, both in education and in the pleasures of life. There are some important points to bear in mind in regard to the care of the ears, but they are not numerous, for, as an American has advised, "Take care of your nose and throat, and trust to God for your hearing." But it is highly import-

THE CARE OF THE THROAT, NOSE, AND EARS IN CHILDREN—*continued.*

ant that the earliest symptoms of deafness in childhood should be recognised. I have little doubt that 90 per cent. of the cases of deafness in adult life can be traced to neglect of this. No child should be labelled as inattentive until the doctor has pronounced that the ears are normal. Even short recurring attacks of very slight deafness and every case of earache should receive skilled medical advice. The wearing of cotton wool in the ear, except when ordered by a medical man, is as unnecessary as it is unsightly, and can only help to make it delicate.

Boxing the ears is so generally recognised as extremely dangerous that it is hardly necessary to refer to it. At the same time it is well to remember that damage to the drum of the ear may occur, if it is weakened by disease, by such acts of the individual as sneezing, coughing, or even blowing the nose. In such occurrences any teacher or nurse who had given a child some mild chastisement might be blamed for an accident to the ear of which they were quite innocent. Injury to the drum of the ear may also occur from other causes than what diplomatists call "unfriendly acts." Even an expression of affection may cause it, as I have heard of the drum being injured from a too vigorous kiss being applied to this orifice.

The ear is kept lubricated by wax. This is secreted in the outer half of the ear passage, and serves a very useful purpose. Like the mucus in the nose, it doubtless acts for the ear as a sort of bird-lime, preventing flies, dust, etc., from getting into the depths of the ear. This wax is being worked outwards by the movement of our jaws in mastication. When it reaches the surface, where it becomes visible, it is wiped away with the ordinary cleaning to the ear. Many people are much too anxious about getting the wax out of their ears. In their efforts to do so they are apt to push it so far inwards that it is no longer worked out by the action of the jaws. Many attempts to extract it may affect the ear drum. Picking out the wax from the ear with pins, toothpicks, ear scoops, etc., should be strictly forbidden. No one should introduce into his or her ear anything smaller than their own little finger, covered with the thickness of a towel.

Children, with their thirst for adventure and the mysterious, frequently introduce foreign bodies into their throats, noses, and ears. The collection is almost too large to enumerate. Perhaps marbles, buttons, beads, seeds, bits of pencil, pens and nails are their favourite treasures. Those in charge of children should be very chary in making hasty attempts at removal. Not infrequently unskilled efforts are only apt to push such a slippery article as a glass bead still further into depths of the ear or nose.

It may be laid down as a principle that no foreign body should be allowed to remain in any of the upper air passages except when under medical inspection. If it is introduced in the nose efforts at expulsion should be encouraged in the method I have described—*i.e.*, *à la paysanne*, as they say in France. For the ear the most suitable remedy is a syringe and some warm water. In the throat no attempt should be made to get rid of a foreign substance, such as a piece of meat, by pushing it backwards, unless the child is threatened with immediate suffocation.

### Incidents in a Nurse's Life.

### THE THANKSGIVING BICYCLE.

"You see, nurse, I quite think the village folk ought to provide you with a new bicycle. You have worn out two of your own and the people are not asked for subscriptions, so I think it is quite time they should do something to help towards the maintenance of a nurse, and I believe that it will give general satisfaction if the Harvest Thanksgiving collection this year should be spent in that way."

Thus the rector of a country parish tried to overcome my objections to his scheme for providing me with a new machine. I felt that I would far rather bear the expense myself than appeal to the villagers. It was true that very small fees were charged to the patients, while non-patients contributed nothing, as the rector himself supplied the nurse's salary. Still I felt distinctly nervous about a public appeal being made. I had found it very uphill work to induce the people to be nursed in a proper manner, and knew that several remarks had been made about the "bothering new-fangled nusses," so, although I had made several staunch friends, I dreaded the "talk" which is part of village life.

However, the following Sunday, on returning from my rounds, I was told that notice had been given during morning service "that the proceeds of the Harvest Thanksgiving collection next Sunday will be used for the purchase of a new bicycle for the district nurse," and the rector had added a few remarks to the effect that the parish generally had profited by her labours during the last six years, etc. In the evening I overtook the party from the rectory on the way to church, and asked the clergyman whether he "would allow me to sit comfortably through the service, or whether he was going to give notice of the collection again?" "I shall certainly speak of the bicycle, but you need not feel uncomfortable," was the reply. Thereupon, I am sorry to confess, I turned on my heel and ignominiously fled home to my cottage, not daring to face the congregation.

During the week after Thanksgiving Sunday I heard various ideas on the subject. Groups of boys as I passed called out, "Well, that bike is the worse for wear," "I say, she do want a new 'un," etc. The general feeling appeared to be that it was quite the right thing to do, and many of the villagers congratulated me most kindly. When calling upon a late maternity patient, the woman said, "I am so glad we are to buy you a new bicycle; we all gave a trifle to it. Georgie was so excited that, before we were outside the church door he called out, "I dave my penny to buy nurse a new bike, 'tos she brought Tommy'" (the new baby). An old man said, "I have not wanted the nurse yet, but none of us know what may happen, and I am pleased enough to help." Another woman remarked, "It will be nice for you to have the new machine; you could not go about so far without one; all my grandchildren put in their pennies and little Charlie said, 'I do want to see the new bike, will nurse let me look at it, do you think?'"

At last the day arrived when I found time to go to the nearest town (eleven miles distant) and make the purchase, leaving my old bicycle behind and riding the new one home. It was a free-wheel, with back and front rim-brakes, and everything suitable to my height and weight. The first time I met "Charlie" I jumped down and told him to admire the glories of the plated rims of the wheels, the handle-bar, etc., allowed him to ring the bell, and finally lifted him to the saddle and gave him a ride, to the great delight of his grandmother, who exclaimed, "There now, Charlie, you have seen the new bike, haven't you?" The whole village admired the machine, and ladies would stop me to ask whether it were less fatiguing to ride a free-wheel, and how many miles an hour I could go, while one lady made me a present of a cyclometer because she was anxious to know how many miles were travelled in a week.

This is now four years ago, and, though it has had new tyres and several small repairs since then, the Thanksgiving bicycle still works well on behalf of my patients, and remains as a constant reminder of the Harvest Festival collection of that year.

### The Nurses of the Incorporation Infirmary, Shirley Warren, Southampton.

### INTERVIEW WITH THE MATRON. BY OUR COMMISSIONER.

THE people of Southampton are justifiably proud of the handsome and commodious building which stands on the heights of Shirley Warren. Erected five years ago by Guardians who desired to provide an institution on thoroughly up-to-date lines and yet to avoid the charge of extravagance, the Incorporation Infirmary, which is entirely independent and far away from the workhouse, is nothing less than a general hospital equipped with every modern appliance. It has also the advantage of standing in admirably arranged and well-planted grounds of several acres in extent, while as to the surroundings, the open-air balconies on the south side face and command a fine view of the New Forest.

However, my object in visiting the infirmary was to make inquiries about the nursing, and on my arrival I was

OUTSIDE THE NURSES' HOME, SHIRLEY WARREN INFIRMARY.

at once shown to the comfortable suite of apartments allotted to the matron, who has also her office in the centre of the building, where she is in touch, by the telephone, with every department. This I soon found out, as Miss Dowbiggin conducted me round, is but one of many well-considered arrangements for saving time, and I was struck by the fact that with the possible exception of the ornate though by no means costly chairs in the Committee-room, there was not a detail to which a fair-minded economist could take exception, even in the interests of the ratepayers. As we proceeded along the airy corridors, with their tasteful colouring, into one of the medical wards, and I referred to the design of the building, the matron mentioned that the architect's success was, perhaps, the more remarkable because it was the first large hospital he had planned. Certainly the construction of the wards, the operating-room, with opaline walls, terrazzo floors, and aseptic dressing-boxes, the useful dispensary, the lofty kitchen, the busy laundry, as well as the smaller rooms, give evidence of the most careful study of the requirements of the times. There is one defect which, happily, can be remedied. The Nurses' Home, in other respects unexceptionable both as to exterior and interior, is a short distance from the infirmary, and there is no covered way. As we returned to the infirmary I asked the matron if the drawback had not been noticed, and she said that it had, though there had not been any complaints from the nurses.

### THE NURSES' HOME.

"Of course," she continued, "it would be better if they could go to and fro under cover, but, as far as possible, the disadvantage has been minimised by the Guardians who have provided snow-shoes and warm cloaks of dark blue serge with scarlet hoods for the nurses to use in wet or severe weather. The absence of a covered way does not apparently affect their health, for we rarely have cases of illness."

"For the rest, the quarters of the nurses seem to be all that is necessary, and I notice that each of the bedrooms has a fireplace."

"Yes, that was made a point of, and I cannot understand why fireplaces are sometimes omitted from nurses' bedrooms, seeing their value from the hygienic point of view, not to speak of the pleasure it gives a nurse who is a trifle indisposed to have a fire in her room. The home contains 30 bedrooms, as well as the sisters' and the nurses' sitting-rooms, the latter having a piano. When enlargement is needed the number can easily be increased."

### POINTS OF THE TRAINING.

"The training school was started when the infirmary was opened?"

"Yes, five years ago next May. It opened with 5 sisters, 10 staff nurses, and 10 probationers. The staff nurses had all left before I came and their places were filled with probationers. I came three years since. I commenced with 28 probationers under new regulations."

"You might mention the nature of some of the regulations."

"The course is for three years, and at the end of that time a certificate is given to the nurses who pass their examination and satisfy the medical superintendent and the matron in their practical work generally. I should like to say that the success of the place is largely due to the medical superintendent, Dr. Bencraft, who spares no effort to help the nurses, and takes a personal interest in all the people here. We have a consulting surgeon, and all operations are done in our theatre. The consulting surgeon is assistant surgeon at the Royal South Hants Hospital. There is one resident medical officer who gives the first course of lectures in elementary anatomy and physiology, and the medical superintendent the second and third courses; while I lecture to them on general nursing work. The assistant matron gives classes in splint paddings, bandaging, and generally assists the nurses in their studies. The theatre sister also lectures to the second-year probationers. There

### THE NURSES OF THE CORPORATION INFIRMARY, SHIRLEY WARREN, SOUTHAMPTON.—*Cont.*

is one point to which I attach great importance. Every three months the ward sisters bring a book to me, showing how the probationers under them have progressed, what they have been taught; they have a syllabus drawn up, but I do not expect them to adhere exclusively to that; they may take any nursing subject and give practical demonstrations in the ward. At the final examination a silver medal is given to the first probationer, and a bronze one to the second."

### The Choice of Sisters.

"How many sisters are there now on the staff?"

"Seven, including the night sister; and there is also an assistant matron, who has charge of the home. The sisters receive a salary of £30 the first year, rising £2 a year to £36. I make a point of getting sisters who are fond of country life, and they all have bicycles. There is no hard and fast rule as to promotion. I am hospital trained, my

Children's Ward, Shirley Warren Infirmary.

school being Leeds General Infirmary. Afterwards I had six months' training at one of the hospitals under the Metropolitan Asylums Board, was then sister at the Royal Portsmouth Hospital, head sister at the Birmingham Convalescent Hospital, and for eighteen months assistant matron of Shoreditch Poor-law Infirmary. My experience convinces me that it is wise to introduce fresh blood sometimes, though if I had a very good nurse I should gladly promote her. But I would sooner have as sister a nurse who had been trained here and had then been for a time staff nurse at a general hospital."

### The Maternity Ward.

"How many nurses are allocated to each block?"

"A block consists of two floors, or 60 beds. The sister in charge has four probationers on day duty and two on night duty. There are 300 beds in the Infirmary, but so far they have never been all filled.

"Your maternity ward seems to be according to approved conditions?"

"At all events, it is, I think, structurally perfect, and the labour-room, as you have seen, is separate from the general ward. We sent up several candidates for the L.O.S. examinations who all passed. We have not yet applied for the certificate of the Midwives Board, but we now average 33 cases a year, and the sister in charge is a trained midwife with the certificate of the Board. The children's ward has 14 cots, and the phthisical ward 30 beds. The patients in the latter have full diet and the open-air treatment.

"Are the hours for the probationers the same as in most large Poor-law Infirmaries?"

"I think so. As to leave of absence the senior probationers get three hours off duty every other day, a half-day once a fortnight, and whole day once a month. The junior nurses have two hours off each day, a half-day once a fortnight, and a whole day once a month. All day nurses are off duty every Sunday, either in the morning, afternoon or evening. Night nurses are off daily from 8.45 to 11.30 A.M., Wednesdays and Sundays from 8.45 to 12.40 and one night every four weeks. The sisters are off one day from 2 to 5, another 6 to 10, and a third 2 to 11; also they have a whole day once a month. All nurses are allowed to breakfast in bed with a fire in their room, which they appreciate, on their day off. All the probationers have a fortnight's holiday. The sisters have three weeks' holiday. The probationers are paid in the three years £10, £12, and £14, but I consider their training is their real payment. Uniform is provided and it is made for them.

### A Money Diet.

"Is there any special feature in the diet?"

"Yes, it is a money diet, not a weight diet. There is much waste in a weight diet, whereas with a money diet there is none. I write out the diet for the week, and the steward executes it, or cuts it down if I exceed the sum allowed. The night nurses have their dinner when they come off duty at 8 A.M., and their breakfast before they go on duty at night, after they have been asleep."

"Have you a weekly diet table?"

"Here is one for the week ending March 13: Thursday—Breakfast, sausages; dinner, beef steak and kidney pies, potatoes and greens, jam sponge roll; supper, liver and bacon. Friday—Breakfast, boiled eggs; dinner, fish, chops, and tomatoes, mashed potatoes, ginger pudding; supper, fish pies, rice pudding or soup. Saturday—Breakfast, boiled ham; dinner, roast mutton, onion sauce, baked potatoes and greens, rice pudding; supper, cold mutton, macaroni cheese. Sunday—Breakfast, haddocks; dinner, cold roast beef, salad, mashed potatoes, apple tarts, custard; supper, cold beef, salad. Monday—Breakfast, fried bacon; dinner, stuffed veal and ham, potatoes and greens, tapioca pudding; supper, porridge and cold veal. Tuesday—Breakfast, kippers; dinner, roast fowls, sausages, potatoes and sprouts, Manchester pudding; supper, hot-pot. Wednesday—Breakfast, fried bacon and

Nurses at the Fire Drill, Shirley Warren Infirmary.

eggs; dinner, roast beef, Yorkshire pudding, baked potatoes, parsnips, baked apple dumplings; supper, soup and cold beef.

### Theatre Parties.

"It only remains for me to ask you about the recreations."

"When the infirmary was opened the Guardians contributed £5 for a library of fiction, several ladies gave books, and now it is self-supporting, the sisters paying a shilling a quarter and nurses sixpence. Medical books are supplied by the Guardians. At times tennis and croquet are played, and there are theatre parties every week. The managers of the Grand Theatre send us four passes to the dress circle for Monday evening, a privilege which affords much pleasure. So far, twenty-five nurses have been trained here since the school was established, and I believe that they have all done well since. Many are in general hospitals; one sister who left here is district midwife in St. Michael's Home, Kimberley. She took her L.O.S. certificate here, and is now training pupils for the Central Midwives Board."

### Lancaster Sanatorium Flooded.

### HOW THE NURSES MANAGED.

On Saturday last the nursing staff at the Corporation Sanatorium, Lancaster, had an unusual experience. It had rained heavily all day and was blowing a gale by night time. About midnight the nurses were startled by a window being blown out in a nurse's bedroom—luckily unoccupied—and the glass door blown in.

About an hour afterwards the matron heard a noise downstairs, and, on looking out of the window, found that the hospital was surrounded by water. At that moment the ward bells rang and she hurried to the wards, to find that the water had suddenly rushed into the wards and was rapidly rising.

Everyone helped the night nurses to remove the patients, wrapped in their blankets, to the first floor of the administrative block, and put them into the beds which the day nurses had just come out of, and by that time the latter were knee-deep in water.

The matron next telephoned to the police and the medical officer of health; then the gas went out, and soon the telephone, too, was cut off. The staff at this point had to rescue the food in the larders, and discovered that it was impossible to stay longer downstairs on account of the strong current of water nearly carrying them off their feet.

After giving the patients warm drinks—fires were all put out by the water and sticks were floating about, but the nurses managed to light the bedroom fires somehow—the staff waited impatiently for daylight. By that time the water in the house had subsided, but it took two hours longer before the matron and her assistants could get through the corridor into the wards. On investigating the latter, they found mattresses floating and the furniture thrown about, the floors raised, and the centre fire-places sunk. The water had been 2 feet 6 inches high in the wards, and, it was said, 6 feet in some parts of the grounds when the tide was at its highest. The Sanatorium was certainly surrounded by 3 feet of water on Sunday, and it is now gradually disappearing. Unfortunately, the administrative block had been newly built and only occupied by the staff for six weeks, and the dado of water and mud has not improved its appearance.

Happily the inmates are none the worse. There was only one enteric case, and the patient took all that happened calmly; while the children suffering from scarlet fever seemed rather to enjoy the excitement. The latter have since been discharged, but the enteric patient is remaining. As the water has to be pumped from the foundations, the wards will not be dry and cannot be repaired at present. The nurses and servants all worked splendidly, and, except that the nice new rooms and furniture are spoiled, no one seems any the worse for the incident.

### The Duchess of Albany at the Royal Free Hospital.

The Duchess of Albany, attended by Lady Evelyn Moreton and accompanied by Mrs. Harkness, visited the Royal Free Hospital, Gray's Inn Road, on Friday afternoon last, when she was received by Dr. Harrington Sainsbury, the senior physician; Mr. Conrad Thies, the secretary; and Miss Cox-Davies, the matron. Her Royal Highness made a careful visitation of the wards, nurses' quarters, kitchens, store-rooms, laundry, etc., and expressed her great satisfaction at the many structural and other improvements recently carried out; also her approval of the proposed erection of two new operating theatres, which are urgently needed to meet the greatly increased surgical work of the hospital. Before leaving her Royal Highness signed the Visitors' Book, and partook of tea in the Matron's sitting-room.

### A Guild of Service for Poor-law Nurses.

A meeting was held at the Church House, Westminster, on Friday to explain the object of the Guild of Service, and to make it more widely known to the class for which it is intended. The Bishop of Kingston was in the chair, and among the speakers were Father Waggett, Miss James (Poor-law Guardian, Bethnal Green), Colonel Barrington Foote, Major Malet, Archdeacon Escreet, and the Rev. H. Westall.

The Chairman said that the object of the Guild was to unite all workers, salaried and voluntary, in Poor-law and kindred institutions in Christian fellowship, and to bring together persons who were like-minded but had not much opportunity of religious worship. He contended that such a Guild was much needed, as in all workhouses and similar institutions there were so many different shades of society that they could only be approached by the touch of humanity and Christian love. The Guild was started with the sanction of the Bishops of the dioceses in which branches had been formed, and he hoped that as the basis of membership was so simple, it would, like the Church of England Men's Society, have a wide success.

Miss Stone, Hon. Secretary for the Southern Province, explained that the Guild had absorbed the Poor-law Chaplains' Association and the Guild of Lazarus, and its first members were admitted in 1904.

Father Waggett spoke warmly of the need of the Guild among Poor-law nurses and workers, who had been hitherto almost forgotten by the Church, and who were cut off from active participation in ordinary parochial life. It was a scheme which would require much real and steady exertion on the part of its members.

Major Malet and Colonel Barrington Foote both dwelt on the power that the Guild might wield, and referred to the splendid work that the Guild of the Holy Standard had done in the army.

Miss James said that she was sure that the public did not realise the number of people who were connected with Poor-law institutions, and what a work this Guild could do among them. There were in England and Wales 20,000 Guardians and 40,000 officers and servants, and so far there had been no special religious organisation which touched their needs, and there was an appalling lack of spirituality in their life. The hopelessness of their work and the bad effect which being in authority over persons of different status had on the character all pointed to a very real opening for the work of the Guild. When one considered that in a Union like Bethnal Green there were 500 deaths yearly, one began to have some conception of the dreariness of a chaplain's life, and the hard battle against despair which every nurse and worker daily had to fight, and she felt sure that the fellowship of the Guild would be a source of strength.

### The Paris Picnic.

### NEITHER INTERNATIONAL NOR PROFESSIONAL.

The republication of the articles on "Make-Believe in British Nursing" has excited a great deal of interest, and we are glad to know that they are being widely and appreciatively read. It is essential, in the best interests of the best nursing, and of professional nurses in the true sense and meaning of this term, that everything connected with British nursing in future shall be undertaken with serious purpose, and solely in the interests of the profession and the trained nurse. In the past, and especially of late, make-believe in British nursing has been so aggressively pursued, that for a multitude of reasons, many of which are widely divorced from the true interests of nurses, the promoters of these masquerades have at length come to regard themselves as the leaders of British nursing. Yet many of them are disqualified from occupying any such responsible position from the fact that they are not at the present time engaged in the teaching and training of nurses, or in the management and control of institutions, through which improved nursing and professional advancement, in the nursing sense, extends and ramifies throughout the Empire. It is for these reasons that it has been necessary to bring out the facts fully and tersely for the information of everybody interested who cares to obtain a copy of the brochure entitled "Make-believe in British Nursing."

### That Official Organ.

It is claimed that the British Islands now possess "an official organ" for nurses, under the professional direction of professional nurses. If this were indeed the case everybody would regret that this official organ, as at present conducted, cannot be regarded as a serious newspaper at all. Its chief aim, if we may judge from its columns, seems to be to emulate, and even to surpass, the yellow Press by its violent, vulgar, unfounded, and therefore ludicrous, attacks upon individuals. The statements of this character published in the "Official Organ," recently, are so misleading as to be untrue and false; or, in other words, they are the worst sort of insinuation—namely, half truths—and come within Shakespeare's saying of the man "who lies two-thirds, and then with a known truth tries to pass a thousand nothings." We are not concerned, and no true journalist is, with individuals, but with principles, and have therefore no intention to retaliate in kind. We regard such procedings with supreme contempt, but it is due to those who are not familiar with the facts that they should be put in a position to ascertain the truth by access to official documents. Such documents are in our possession and may be inspected under necessary safeguards.

### The Incontrovertible Facts.

The important point, however, for the whole nursing profession, and for every individual nurse who is zealous for the honour of her profession to grasp is, that not one single fact contained in our exposure of The Stage Army has been controverted or contradicted. Abuse of individuals is no answer, and it is well that this point should be understood by every trained nurse. She will then realise that in fact there is at the present time no International Council of Nurses as such, in any representative sense. What a farce it is, too, to make all this stir about professional journalism, and the exclusion of the laity and the doctor from the councils of the professional nurse, when the promoters of the Paris gathering have thrown open the meetings to the lay public as well as to nurses and members of the medical profession! The absurdity and hollowness of the whole claim could not be more strikingly displayed than in the announcement that a discussion, in which the lay public is invited to take part, will be opened by Mrs. Bedford Fenwick and others on "Professional Organisation: The History of the Professional Nursing Press." Have these estimable ladies no sense of humour? Are they so deadly in earnest that they have persuaded themselves that vindictiveness is force, abuse argument, and that laughter cannot be tolerated by the leaders of The Stage Army and its masquerades?

### Catholic Nurses' Association.

The Catholic Nurses' Association was formed ten years ago, its birthplace being the Convent of the Visitation at Harrow-on-the-Hill. Its object is similar to that of the Guild of St. Barnabas—to bring Roman Catholic nurses together for mutual and spiritual help. There are at present 100 members, but it is hoped to increase the number by making the Association better known.

The monthly meetings were originally held at the Convent of the Visitation, but it was found that many of the nurses working in London hospitals could not get so far in their off-duty time. It was therefore arranged for the meetings to take place alternately at 109 St. George's Road, Southwark, and at 15a Vicarage Gate, Kensington. The sisters at Harrow still take a warm interest in the Association, and members will always find a warm welcome whenever they can find their way to the Convent.

The house at Vicarage Gate is a Residence for Working Girls, and it was in the big committee-room which is used for meetings, dances, and gatherings of all sorts in connection with the Catholic Settlement, of which the home is a branch, that the last Conference under the auspices of the Association was held on Thursday last week. About twenty-five members were present. Father Galton, of Farm Street Chapel, conducted, and, after an address from him on the special subjects of Lenten meditation and preparation, the company adjourned to the little chapel, where Benediction was sung, returning for a pleasant tea and talk in the committee-room.

The next Conference will be held on April 18, at 109 St. George's Road, Southwark, opposite the Cathedral.

### Everybody's Opinion.

### A PROBATIONER LOSES HER SIGHT.

"Policy Holder No. 7350, Royal National Pension Fund for Nurses," writes : I enclose postal-order for 15s. for a nurse who lost her eyesight in nursing a typhoid patient. 10s. is from a friend and 5s from myself.

### A LONG-FELT WANT.

"E. S.," Abingdon, writes : I think "Puzzled One" would find a pair of instep-arch socks, inside ordinary felt slippers, quiet and comfortable, and meet the difficulty. There are some such socks advertised in the *Nursing Mirror* of March 9. I had a pair from Thos. Holland and Son, 46 South Audley Street, Grosvenor Square, W. They are quite good now, although I have used them a long time. The price was 5s. 6d.

"A Kilburn Nurse" writes : Your correspondent "Puzzled One" inquires for noiseless night-shoes with heel. She can obtain kid shoes, with felt soles and heels, at the Army and Navy Stores, Victoria Street, Westminster, price 6s., and for a small additional charge they can be made to measure.

"Old Westminster" writes to the same effect, and she adds : These shoes are quite presentable for day-wear while new, and they stand hard night-wear for a long while. I find Jaeger shoes (with rubber heels extra) almost silent; they are 7s. 6d. Both are well worth the money in the long run.

### IN SEARCH OF A DOCTOR.

"C.M.B." writes : Having been a midwife attached to one of the large London maternity hospitals, I am absolutely amazed at the letter from a "District Midwife" in your issue of March 9. Her ignorance of the Act is astounding. I should like to know what right she had to attend a case of four months' miscarriage, and then she tells us she wishes she had been called in sooner. Does she mean to say she would have medically advised and treated a pregnant woman suffering from hæmorrhage under four months? Can she wonder the doctors refused to come? What doctor would care to take over for 10s. 6d., or, indeed, any fee, a case of a four-months' miscarriage where an examination had been made by a midwife? And when a midwife sends for a doctor a written note should be sent, not a message; and may I suggest that it was a fœtus, not a "baby" the "District Nurse" delivered her patient of. By telling us it was "dead" I fear that she labours under the impression it might have lived had a doctor arrived in time.

### THE DISTRICT NURSE AND THE DOCTOR.

"Sincerity" writes : I am glad to add my testimony to that of "Justice," *re* District Nurse and Doctor. I, too, have worked in many parts of London as district nurse, and I have always had the greatest kindness and consideration from the doctors, and many of them have been my best friends, and are still. I am afraid that the fault is often with ourselves if we do not get treated kindly. May I give you an instance of how one of the kindest and best doctors has recently been treated by a district nurse. A message was sent to a certain doctor to call on Nurse ——. By a mistake the message was delivered to the wrong doctor, who came almost immediately. When the nurse looked up and saw it was not the one she had sent for she simply turned her back on him and even refused to answer his "Good morning, Nurse." Can we wonder at some doctors looking with disfavour on the district nurse? Most of the doctors treat us as fellow-workers with themselves; may we be worthy of the honour.

### POOR-LAW INFIRMARY-TRAINED NURSES.

"Bucks" writes : I would like to say that I also have often wished to ask the same question, as I feel the slight put upon us. A few weeks ago I saw an advertisement in The Mirror from a superintendent of a district nurses' home, which said : "No Union nurse need apply." And yet I, who am workhouse trained, was the first district nurse in that large manufacturing town, and I say, without any pride, that it was owing to the success attending my work that a large body of persons met to form an association. I was offered a post there, but I continued for about eighteen months longer as nurse under the same committee with which I had started, as I did not want to join a nursing home. I then left, against the wishes of my committee, as I saw how much better it would be for the whole town to be united. The same superintendent who is now advertising told my committee, when I passed over my cases to them, that they were a credit to me, they were so beautifully clean. Those words have always remained with me, though said so very many years ago. Since then I have worked for two other districts, and have frequently been thanked by the surgeons after operations in small houses for my help. So I hope that "Hurt" will take courage, as I have done. It is the patient and doctors who know the worth of Union-trained nurses. My experience, after sixteen years' nursing, points to the fact that in the district we are more successful than hospital nurses. I have never yet failed to be able to follow the doctors' instructions, and many nurses who were trained with me have joined the Queen's Nursing Homes in very large centres. There are only a few superintendents and matrons who object to us, simply because they do not understand our capabilities. As we get older one finds that more is needed than an over-abundance of theory. I would rather be told that I had made a patient comfortable than that I was clever.

### Appointments.

Bolingbroke Hospital, Wandsworth Common, London. Miss Gertrude Pullen has been appointed sister. She was trained at St. Thomas's Hospital, and has since been staff nurse at Bolingbroke Hospital.

Edinburgh Royal Infirmary.—Miss Annie Warren Gill has been appointed lady superintendent of nurses. She was trained at the Royal Infirmary, Edinburgh, where she was afterwards sister, night superintendent, and assistant lady superintendent in charge of the Nurses' Home. She served in South Africa as matron of the Edinburgh and East of Scotland Hospital, and was afterwards one of the matrons in the Concentration Camps, Orange River Colony, receiving for her services during the war the decoration of the Royal Red Cross. She has since been matron of the Royal Berkshire Hospital, Reading.

Egham Cottage Hospital.—Miss E. Morretti has been appointed staff nurse. She was trained at Bolingbroke Hospital, where she has since been staff nurse.

Gravesend Borough Sanatorium.—Mrs. L. Scott has been appointed matron. She was trained at the Royal Infirmary, Newcastle-on-Tyne, and has since been charge nurse at Eaton Hospital, matron of Eaton Sanatorium, and matron of Harrogate and Knaresborough Joint Isolation Hospital.

Huddersfield Sanatorium.—Miss Bellona Watkins has been appointed sister. She was trained at the City Hospital, Coventry, and at the Royal Infirmary, Sheffield.

London Homœopathic Hospital.—Miss Clara Hoadley has been appointed matron. She was trained at Guy's Hospital, and holds its silver medal. In December 1898 she was appointed assistant matron of the Royal Infirmary, Preston, and she has since been lady superintendent and matron of the Royal National Hospital for Consumption in Ireland and Matron of the Coventry and Warwickshire Hospital.

Lynton Cottage Hospital, North Devon.—Miss A. Watson has been appointed matron. She was trained at Nuneaton Hospital, and has since been holiday sister in charge of Leeds City Fever Hospital, nurse-matron at

Gately Cottage Hospital, and district nurse at Crewe. She has also done private nursing.

Royal Halifax Infirmary.—Miss E. S. Innes has been appointed assistant matron. She was trained at Leeds General Infirmary, where she has since been ward sister, sister in charge of theatre, and night superintendent.

Shirley Warren Infirmary, Southampton.—Miss Dorothy Trinder has been appointed sister. She was trained at Shoreditch Infirmary, London, where she has since been staff nurse. She holds the C.M.B. certificate.

Shoreditch Infirmary.—Miss Daisy M. Cordingley has been appointed sister and Miss Nellie Blackburn maternity sister. Miss Cordingley was trained at the General Hospital, Perth, Australia, and has since been staff nurse at Albany Hospital, Perth, Australia, staff nurse at St. Luke's Home, Bayswater, London, and charge nurse at the Park Fever Hospital, London. Miss Blackburn was trained at Shoreditch Infirmary, where she has since been staff nurse. She holds the C.M.B. certificate.

Southwark Infirmary.—Miss E. Howell Bridger has been appointed sister. She was trained at Southwark Infirmary.

Stourbridge Union Infirmary.—Miss Elsie Price has been appointed superintendent nurse. She was trained at Brownlow Hill Infirmary, Liverpool, and has since been sister of male wards at King's Norton Infirmary, and superintendent nurse at Solihull Infirmary.

Wandsworth Union Infirmary.—Miss Helen Todd has been appointed matron. She was trained at St. Bartholomew's Hospital. She has since been assistant matron at the Hackney Union School at Brentwood and matron of the Royal National Sanatorium, Bournemouth.

### Queen Victoria's Jubilee Institute for Nurses.

Miss Louisa J. Attree has been appointed to Enfield; Miss Lois Dawson, to East London; Miss Ernestine Edgcumbe, to Gower, South Wales; Miss R. Knight, to Warrington; Miss Jean Neilson, to Southborough; and Miss Kathleen Rogers, to Crook. Miss Minnie Jarvis has been transferred to Woolwich from Gosport; Miss Elizabeth Monro has been appointed temporarily to Coventry as superintendent; and Miss K. B. Harrison temporarily to Heanor as nurse.

### Presentations.

Nurses' Co-operative Home, Dublin.—An interesting event took place on Saturday at No. 86 Lower Leeson Street, when Miss M. E. MacDonnell, late superintendent of the Nurses' Co-operative Home, was the recipient of an illuminated address and a handsome medical electrical battery presented to her by the nurses of her staff as a token of their regret and good wishes on her departure to India. The nurses present having expressed their sorrow to Miss MacDonnell at her leaving them, the latter replied in suitable terms.

### Novelties for Nurses.

(By our Shopping Correspondent.)

### A SELF-FILLING PEN.

In these busy days a stylographic pen of some sort is an absolute necessity. Both men and women who have much to do cannot possibly manage without one, and the very schoolboys and schoolgirls of this generation find it hopeless to get into the Honours List, when the examination paper is long, if they do not carry their own faithful "stylo" with them. But how many of us have started to scribble a letter or to write out a lecture in the few minutes at our disposal and found, to our vexation, our pen empty and no filler at hand? At last a pen has come from the United States (London agents, American Agencies, Ltd., 38 Shoe Lane, E.C.) which feeds itself. It is called "The Conklin Self-Filling Pen." There is nothing elaborate about it; one turn of a ring, one pressure of a metal rib, and the pen is ready again for use in less than a moment. It is quite easy to clean and to keep in order; it cannot roll off the desk in the aggravating way of most fountain-pens; every part which wears out can be replaced at a fixed charge; and the price, including a gold nib, starts at 12s. 6d. Any nurse who buys one of the "Conklin Self-Filling Pens" on this recommendation will not regret the expenditure.

### TRAVEL NOTES AND QUERIES.

By our Travel Correspondent.

Switzerland for Two or Four Weeks (H. B.).—I shall be pleased to help you, when I have any information to work on. Please write again and tell me exactly how much you can each afford to spend inclusive of the expenses of journey. When I know this I can map out a tour. Why do you wish to travel first class? It is quite unnecessary, and will make a very serious difference in your expenses. The journey is not long, and second class is very comfortable. I should not advise cycles; for inexperienced travellers they are a nuisance.

A Month in the Pyrenees (A. W.).—Write to me again later on; you cannot go to the Pyrenees till May. If it is imperative that you should be abroad in April, Biarritz would be more suitable; on this point I do not quite understand your letter. Write me again more clearly.

The North of Ireland (A Constant Reader).—You do not tell me where you wish to go in Ireland, but for the North, Belfast would be best. Third-class return will be about £2 5s. Certainly you can have a return ticket now. Write to the Traffic Manager at St. Pancras, enclosing a stamped and addressed envelope, asking him to tell you the exact cost of a third-class return tourist ticket; it is possible it may be a trifle less than the sum I quote. Remember these figures only relate to the journey between London and Belfast. If you do not like to travel steerage on the boat (the passage is six hours) you can by paying 10s. 3d. extra, cross in the saloon both ways. It is worth while if you are a bad sailor.

A Quiet Address in Paris (Paris).—I fear there is nothing quite so cheap as you desire. If I knew the object of your visit I could perhaps help you more. Try the Girls' Friendly Society, 17 Rue Cour Celles; if you can get in, your terms would be accepted. Write to Mrs. Collyer, the Lady Superintendent on a prepaid foreign return postal card, and ask her if you are eligible for entrance. Write in the same way to Madame Gilbert, 24 Rue Davioud Passy (this is a Home for Governesses). Also try the British and American Mission Home, 77 Avenue de Wagram, and Madame Wellington, 61 Rue Spontini, Avenue Victor Hugo. If you do not succeed with any of these write again, and give more particulars.

Rules in Regard to Correspondence for this Section.—All questioners must use a pseudonym for publication, but the communication must also bear the writer's own name and address as well, which will be regarded as confidential. All such communications to be addressed "Travel Correspondent, 28 Southampton Street, Strand." No charge will be made for inserting and answering questions in the inquiry column, and all will be answered in rotation as space permits. If an answer by letter is required, a stamped and addressed envelope must be enclosed, together with 2s. 6d., which fee will be devoted to the objects of "The Hospital" Convalescent Fund. Ten days must be allowed before an answer can be published.

### Notes and Queries.

**REGULATIONS.**

**The Editor is always willing to answer in this column, withou any fee, all reasonable questions, as soon as possible.**

**But the following rules must be carefully observed.**

**1. Every communication must be accompanied by the name and address of the writer.**

**2. The question must always bear upon nursing, directly or indirectly.**

**If an answer is required by letter a fee of half-a-crown must be enclosed with the note containing the inquiry.**

*Artificial Limbs.*

(238) Is there any society which gives artificial limbs to the poor? A girl who has lost both legs is in need of such.—*Somerset.*

If the congregation to which the girl belongs contributes to the London Hospital Sunday Fund, apply to her Vicar and he may be able to help her. The Surgical-Aid Society, Salisbury Square, Fleet Street, E.C., supplies limbs, but letters are necessary.

*Maternity Nursing.*

(239) I am a trained nurse. Is there any institution where I can train in maternity nursing? I cannot afford a large fee.—*Sophy.*

Nearly all the lying-in hospitals and the Maternity Nursing Association, 63 Myddleton Square, E.C., receive trained nurses at reduced fees. Write for particulars.

*Children's Nurse.*

(240) I should like to train as a children's nurse. Is there any children's hospital which teaches?—*Ignorant.*

There are special institutions for this special training, as, for instance, the Liverpool Ladies' Sanitary Association, 27 Leece Street, Liverpool; The Princess Christian's Training College, 19 Wilmslow Road, Withington, Manchester; and the Training Home, Flora Villa, The Wrythe, Carshalton, Surrey.

*Training in Midwifery.*

(241) Have boards of guardians and district councils powers to train and employ midwives?—*E. L. O. E.*

Boards of guardians and district councils have, we believe, power to pay for midwifery training for women whom they wish afterwards to employ as midwives, but no woman can now be a midwife unless she has passed the examination of the Central Midwives Board.

*Nursing in India.*

(242) I wish to join the Indian Nursing Association; from whence can I obtain information?—*Westminster.*

Write to the Secretary, Indian Nursing Association, Simla; but at present all vacancies are filled up.

*Probationer.*

(243) I am anxious to enter a hospital as probationer. Is it correct for me to apply to the hospital I select, or should I advertise?—*Sarum.*

Apply direct to the hospital.

*Education.*

(244) Will you give me the address of a hospital where knowledge of reading, writing, arithmetic, and composition is necessary?—*A Lover of Nursing.*

Such knowledge is required at every hospital. For a list get "How to Become a Nurse," price 2s. 4d. post free, from The Scientific Press, 28 and 29 Southampton Street, Strand, London, W.C., or from a bookseller.

*Training as a Nurse.*

(245) *Hopeful,* writing from Blackheath, has not sent her name with the query. On receiving it we will do our best to help her.

**Handbooks for Nurses.**

| | Post Free. |
|---|---|
| "A Handbook for Nurses." (Dr. J. K. Watson) ... | 5s. 4d. |
| "How to Become a Nurse." (Sir Henry Burdett) ... | 2s. 4d. |
| "Nurses' Pronouncing Dictionary of Medical Terms" | 2s. 0d. |
| "Art of Massage." (Creighton Hale) ... ... ... | 6s. 0d. |
| "Surgical Bandaging and Dressings." (Johnson Smith.) ... ... ... ... ... ... ... | 2s. 0d. |
| "Surgical Instruments and Appliances Used in Operations," illustrated. (Harold Burrows, F.R.C.S.) | 1s. 8d. |
| "Hints on Tropical Fevers." (Sister Pollard.) ... | 1s. 8d. |

Of all booksellers or of The Scientific Press, Limited, 28 & 29 Southampton Street, Strand, London, W.C.

### For Reading to the Sick.

LIFE'S GETHSEMANE.

GETHSEMANE! Joy hath not flowers so sweet
As those which cluster on thine olive slope:
Beneath the crimson sheen of Jesu's Feet,
Springs up the blossom of a deathless hope.

Oh, not as I but as Thou wilt, my Lord,
I will not put aside Thy cup of pain:
Sorrow is turned to gladness at Thy word,
And Life's Gethsemane becomes a gain.

*Alan Brodrick.*

"Even so, Father: for so it seemed good in Thy sight" (Matt. xi. 26).

The most humble, the most holy, the most true sentence that was ever uttered in this world! I should place it higher than "Not My will, but Thine, be done." For there, there is still a recognition of a will, though it be subdued—a victory, but a struggle. But here it is just what we want: not to have no will—that would not be human; not to have a will conquered—that is not enough; but to have it absorbed. "Even so, Father: for so it seemed good in Thy sight."—*Anon.*

Observe how much easier it is to see and feel God's Hand in some kinds of sorrow, especially the graver troubles of life, than it is in others. The greatest neglect in the opportunity of spiritual growth, presented by suffering, is in the matter of the small daily trials and vexations, which constitute a most important element of the Divine discipline and education of our souls. Are they not, one and all, drops of the Sacramental Cup of affliction, appointed by our Father's love for our strengthening and purification? It is just their humiliating littleness that adapts them most perfectly to His holy purposes. It is, perhaps, in these that our main probation lies. These represent the finest, lightest touches of the Heavenly Sculptor upon the marble of the soul, which He is fashioning towards the Image of His Son. The great sorrows have their special work to do; but the lesser pains and trials, the weariness of waiting when the pain itself is stilled, the wounds of unkindness, the burden of inevitable anxieties and cares—these hidden troubles have their own peculiar worth and service for the great Sculptor; they finish the work of God, and add peculiar graces of refined expression to the soul's "character."—*E. B. Ottley.*

Oh! those who have known the blessings of sickness can never murmur under its appointment, for then it is God makes us "taste and see" how gracious He is, and how blessed it is to trust in Him; and though He lays us low with one hand, yet with the other He holds us up, and we feel around and beneath us the everlasting arms, embracing, comforting, supporting us, yea, cherishing us as a nurse her children. Oh then, then it is that the troubled soul returns to its rest! It has wearied itself flying up and down seeking rest; but this world is only covered with the unsatisfying waters of vanity, and can give none, and so it returns to God.—*M. B. B.*

## Figures and Tables

**Figure f1:**
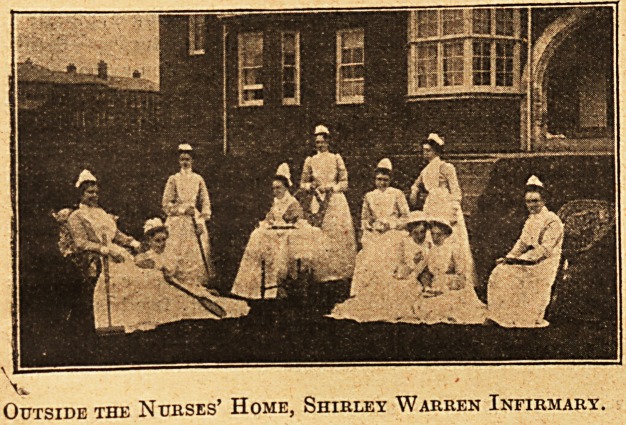


**Figure f2:**
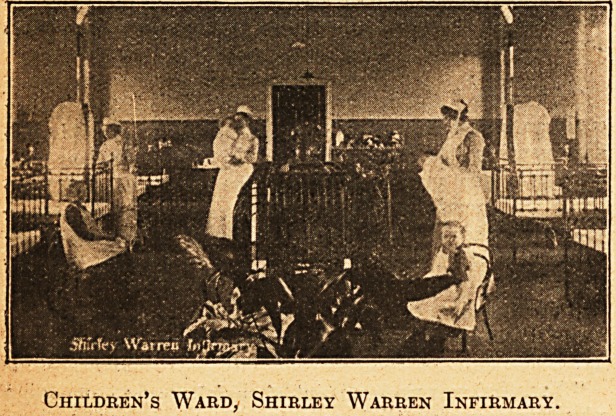


**Figure f3:**